# Validation of gallbladder absorbed radiation dose reduction simulation: human dosimetry of [^18^F]fluortriopride

**DOI:** 10.1186/s40658-018-0219-6

**Published:** 2018-10-08

**Authors:** Robert K. Doot, Jacob G. Dubroff, Joshua S. Scheuermann, Kyle J. Labban, Jenny Cai, Chia-Ju Hsieh, Shihong Li, Hsiaoju Lee, Erin K. Schubert, Catherine Hou, Regan Sheffer, Alexander Schmitz, Kuiying Xu, Robert H. Mach

**Affiliations:** Department of Radiology, Perelman School of Medicine, University of Pennsylvania, Philadelphia, PA 19104 USA

**Keywords:** PET, [^18^F]fluortriopride, Dopamine, Dosimetry, Gallbladder absorbed dose

## Abstract

**Background:**

[^18^F]Fluortriopride (FTP) was developed as a dopamine D3-selective radiotracer, thought to be important to neurobiological reward pathways and implicated in drug addiction, Parkinson’s disease, and schizophrenia. Preclinical radiation dosimetry studies found the gallbladder wall received the highest dose. A gallbladder dose reduction intervention was simulated using a novel reduction model for healthy adults following fatty-meal consumption. The goals of this study were to assess whole body FTP human dosimetry and determine the feasibility of reducing absorbed dose to the gallbladder wall.

**Results:**

Effective dose without a fatty meal was 0.022 ± 0.002 mSv/MBq (± standard deviation) with highest organ dose of 0.436 ± 0.178 mSv/MBq to the gallbladder wall (*n* = 10). Predicted gallbladder dose reduction with fatty meal consumed was 67.4% (*n* = 10). Meal consumption by four repeat volunteers decreased average gallbladder dose by 71.3% (*n* = 4) compared to the original ten volunteers.

**Conclusions:**

Observed effective doses were adequately low to continue studying FTP uptake in humans. Validated dosimetry simulations indicate up to a 71% reduction in gallbladder dose can be achieved by employing intrinsic physiology to contract the gallbladder via fatty meal ingestion. This methodology for predicting gallbladder absorbed dose reduction from fatty meal consumption can be applied to other radiopharmaceuticals and radiotherapies.

## Background

Dopamine D3 receptors are thought to be the principal neural substrate in the dopaminergic mesolimbic circuit, the primary reward pathway in the mammalian brain. D3 receptor dysfunction has been implicated in many diseases including drug addiction, Parkinson’s disease, and schizophrenia [1–4]. Selective imaging of dopamine D3 receptors may reveal their role in diseases and could lead to more effective D3-specific therapies for addiction cessation [5–9] and schizophrenia [10]. Until now, determining the role of D3 receptors in addiction and relapse has not been feasible, as current radioligands ([^11^C]raclopride [11], [^18^F]fallypride [12], and [^11^C]PHNO [9]) are not sufficiently selective for distinguishing D2 versus D3 receptor subtypes [12–14]. In response to this need, the Mach laboratory developed [^18^F]fluortriopride (FTP), a D3-specific radiotracer having a D3:D2 selectivity of 163 [15, 16].

Preclinical radiation dosimetry studies of FTP in nonhuman primates identified the gallbladder wall as the critical organ, potentially limiting use in human subject research [17]. In 21 Code of Federal Regulations 361.1 for radiopharmaceuticals regulated by local Radioactive Drug Research Committees (RDRC), the FDA sets research subject single organ radiation exposure limits of 50 mSv (5 Rems) and 150 mSv (15 Rems) for a single dose and annual total dose commitment, respectively [18]. While these limits do not currently apply to FTP because it is regulated under an approved exploratory Investigational New Drug (xIND) application and therefore directly monitored by the FDA, the RDRC limits are often used by local radiation safety committees as guidelines for proposed research. The goals of this study were to assess human biodistribution of radiation doses resulting from administration of the novel FTP radiotracer and to evaluate an intervention to reduce radiation dose to the gallbladder in order to improve subject safety and facilitate serial positron emission tomography /computed tomography (PET/CT) scans.

## Methods

### Volunteers

This study was conducted in accordance with the Declaration of Helsinki, and all procedures were approved by the University of Pennsylvania Institutional Review Board (FWA00004028) and the ClinicalTrials.gov Identifier is NCT02379338. The control group consisted of ten healthy adult volunteers who underwent series of PET whole body and low dose, CT attenuation scans following the intravenous administration of FTP. A simulation group was later created, made up of the simulated change in biodistribution due to fatty meal consumption at 90 min, for each of the ten participants in the control group. The intervention group, consisting of four of the original control group subjects, repeated their dosimetry studies with the additional consumption of a fatty meal supplement. The patients provided signed informed consent for all of the PET/CT studies.

### Imaging protocol

All studies were performed using a Philips Ingenuity TF PET/CT scanner (Philips Healthcare, Cleveland, OH, USA). This scanner has an 18-cm axial FOV and a 70-cm-diameter gantry aperture and a PET spatial resolution of 4.8 to 5.1 mm full width at half maximum, sensitivity of 7.3 cps/kBq, and peak trues rate of 365 kcps [19]. For image reconstruction, the time of flight information along with physical data corrections (e.g., scatter and attenuation) are included in the system model of the list-mode, blob-based, ordered subsets maximum likelihood expectation maximization algorithm (19). Attenuation correction and anatomical registration were both provided by the 64-slice Ingenuity CT scanner (Philips Healthcare), equipped with iterative image reconstruction for low dose CT imaging.

Each volunteer received seven whole body PET scans and up to four low dose CT attenuation scans over approximately 4 h, after fasting for a minimum of 4 h to minimize differences in endogenous dopamine levels in participants due to consumption of food. During the first approximately 90 min, four whole body scans were completed immediately following one another, starting at approximately 2 min post-injection of FTP. The length of each scan depended on the subject’s height and the number of fields of view needed to cover from the top of the head to mid-thigh. Between the fourth and fifth scans, each volunteer was provided an opportunity to urinate with collection of urine. Additional PET/CT whole body scans were done starting at approximately 120, 180, and 220 min post-injection. A second opportunity to urinate with collection of urine was offered after the final imaging session. The time of collection and total volume collected for each urination were recorded, and urine was assayed for radioactivity.

### Dosimetry calculation

The total activity residing in relevant organs for each time point was determined from volumes of interest (VOI) measurements of the PET images using Pmod v3.7 image analysis software package (PMOD Technologies Ltd., Zurich, Switzerland). Individual VOIs completely encompassed each patient’s brain, gallbladder, heart contents, heart wall, intestines, kidneys, liver, lungs, spleen, and urinary bladder to measure total activity by organ. The total activity and volume in five manually drawn lumbar vertebrae marrow spaces were measured to estimate the total activity in red bone marrow by assuming a red marrow density of 1.03 g/cc [20] and weight equal to 1.6% of total body weight [21]. The resulting total activity for each organ was converted into percent injected activity residing in each organ at each time point and entered into the exponential modeling module of the OLINDA | EXM v1.1 software [22]. For organs where the exponential modeling module was a visibly poor fit to the time activity curve, a trapezoidal Riemann sum was used to calculate the number of disintegrations occurring during the PET imaging time points. For the Riemann sums, after the last imaged time point, physical decay was assumed to be the only method of clearance, and the number of disintegrations from the last time point to infinity was calculated as the definite integral of the radioactive decay function. The cumulated activity for each organ was used as the input for the dosimetry estimates in OLINDA | EXM. All dose estimates were calculated using the Standard Adult Male phantom in OLINDA.

### Gallbladder dose reduction model

**Fig. 1:**
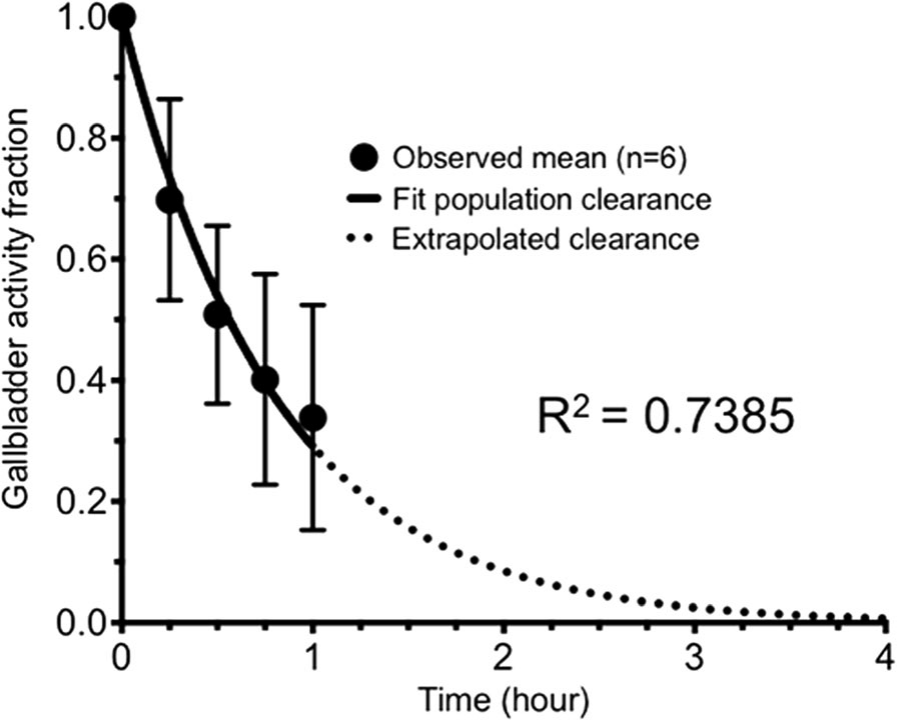
Population gallbladder clearance curve for healthy adults with standard deviation error bars

A gallbladder dose reduction model was developed after preliminary results from preclinical studies indicated the gallbladder wall was the critical organ [17]. The goal of the model was to predict individual gallbladder wall doses assuming gallbladder contraction would follow consumption of a fatty meal. The time of fatty meal ingestion for the simulation was set to 90 min after injection of FTP, based on the anticipated end of future dynamic FTP PET imaging sessions. A fatty meal supplement-stimulated population gallbladder clearance curve for healthy adults was derived using gallbladder time-activity curves from six healthy humans (4 females and 2 males) who broke a ≥ 4-h fast by drinking an 8 oz can of Ensure Plus in a Ziessman et al. [^99m^Tc]Mebrofenin cholescintigrapy study [23]. This data was normalized by peak uptake, and the results were averaged to yield a population gallbladder clearance curve for healthy adults breaking a ≥ 4-h fast by consuming a fatty meal as shown in Fig. 1. The population gallbladder clearance was fitted using GraphPad Prism 7 (GraphPad Software, La Jolla, CA, USA) to yield the following:

1 $$ A={A}_o{e}^{-1.23t} $$

where *A* is the estimated gallbladder activity, *A*_*o*_ is the initial gallbladder activity at time of fatty meal consumption, and *t* is the time in hours after consumption of fatty meal. The number of disintegrations occurring after consumption of a fatty meal can be simulated using the integral of Eq. 1 from time zero to infinity:

2 $$ \overset{\sim }{A}=0.813{A}_o $$

where $\overset{\sim }{A}$ is the cumulated activity in units of Bq s. The total number of disintegrations for a fatty meal-stimulated imaging protocol was then calculated for each individual by adding $\overset{\sim }{A}$ to the Riemann sum from the scan start to 90 min. The simulation’s reduction in gallbladder disintegrations was added to the corresponding individual’s intestine disintegrations. The individual simulated gallbladder and intestinal number of disintegrations were then substituted for the corresponding individual control cohort values as input for the simulation dosimetry estimates in OLINDA | EXM. After model results indicated a large reduction in gallbladder dose, the intervention group repeated their previous dosimetry studies with the additional consumption of a fatty meal supplement (8 oz can of Ensure Plus; Abbott Laboratories) to validate the model.

### Statistical methods

Statistical analyses were conducted using Microsoft Excel (Microsoft Corporation, Malvern, PA, USA) for mean, standard deviation, Reimann sum, and Student’s *t* tests. Graphical analyses of groups used mean and standard deviation values. GraphPad Prism 7 software was used for all scientific graphing presented in this study. IBM SPSS (IBM, Armonk, NY, USA) was used for analysis of variance (ANOVA) and associated Tukey honest significant difference (HSD) post hoc tests to determine specific between-group differences. *P* values less than 0.05 were considered significant.

## Results

### Volunteer characteristics

Ten healthy adult volunteers in the control group (2 females and 8 males, ages 21–53, mean 32 ± 9.6 (± standard deviation)) underwent PET/CT attenuation scans following the intravenous administration of FTP (mean 240.5 MBq, 159–270 MBq (6.5 mCi, 4.3–7.3 mCi)). The simulation group’s characteristics matched the control group because each subject in the simulation group was based on one subject in the control group. Four volunteers recruited from the control group made up the intervention group (1 female and 3 males, ages 29–39, mean 33 ± 5.3) and underwent repeat FTP dosimetry studies (mean 234 MBq, 210–258 MBq (6.3 mCi, 5.7–7.0 mCi)) with the addition of a fatty meal consumption at approximately the same time used for the simulation group.

### Control group results

The FTP average absorbed dose estimates for the control group by region are shown in Table 1.

**Table 1:** Mean FTP radiation dose estimates for the control group (mSv/MBq, *n* = 10)

Target organ	Mean	Standard deviation
Adrenals	1.48E−02	1.36E−03
Brain	4.77E−03	9.51E−04
Breasts	6.06E−03	7.53E−04
Gallbladder wall	4.36E−01	1.77E−01
Lower large intestine wall	3.38E−02	8.95E−03
Small intestine	8.68E−02	2.54E−02
Stomach wall	1.32E−02	1.19E−03
Upper large intestine wall	1.00E−01	2.91E−02
Heart wall	1.63E−02	2.85E−03
Kidneys	2.73E−02	3.47E−03
Liver	6.50E−02	1.05E−02
Lungs	2.70E−02	1.31E−02
Muscle	8.41E−03	3.01E−04
Ovaries	1.99E−02	3.88E−03
Pancreas	1.79E−02	2.32E−03
Red marrow	1.52E−02	7.40E−04
Osteogenic cells	1.28E−02	7.21E−04
Skin	5.15E−03	2.56E−04
Spleen	3.76E−02	3.47E−02
Testes	5.43E−03	2.80E−04
Thymus	6.81E−03	9.35E−04
Thyroid	5.32E−03	5.87E−04
Urinary bladder wall	1.91E−02	5.15E−03
Uterus	1.66E−02	2.74E−03
Total body	1.15E−02	5.50E−04
Effective dose equivalent	5.55E−02	1.12E−02
Effective dose	2.25E−02	2.20E−03

**Fig. 2:**
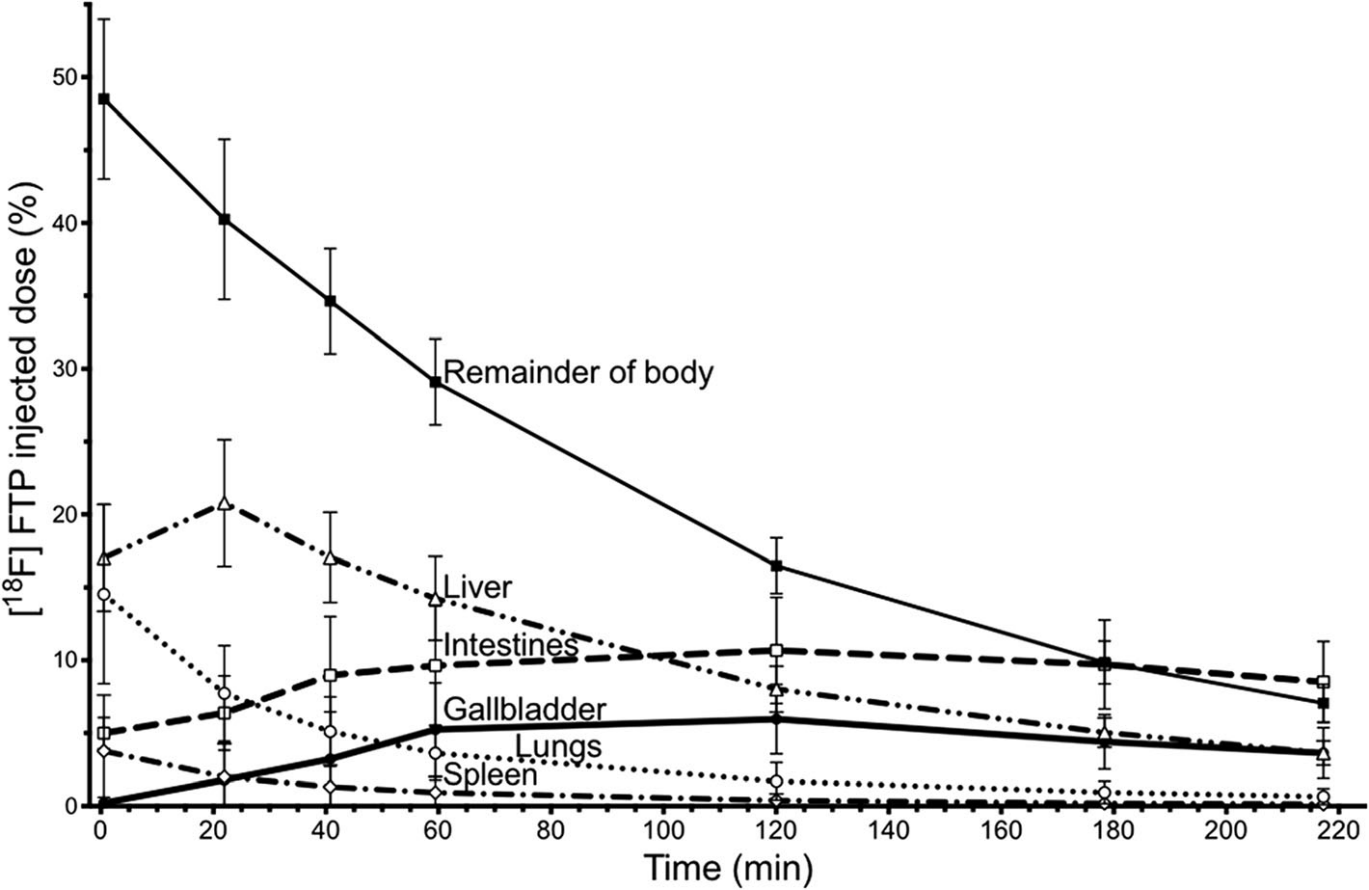
FTP percent injected dose measures over time for the control group

The highest individual organ dose in the control group is in the gallbladder (0.436 ± 0.117 mSv/MBq), which results in a dose of 105 mSv with an injection of 241 MBq. The change in percent injected dose over time for the regions with the highest FTP uptake are shown in Fig. 2. We subsequently simulated and tested an intervention to reduce the high dose in the gallbladder wall to improve future participant safety.

### Simulation and intervention group results

**Fig. 3:**
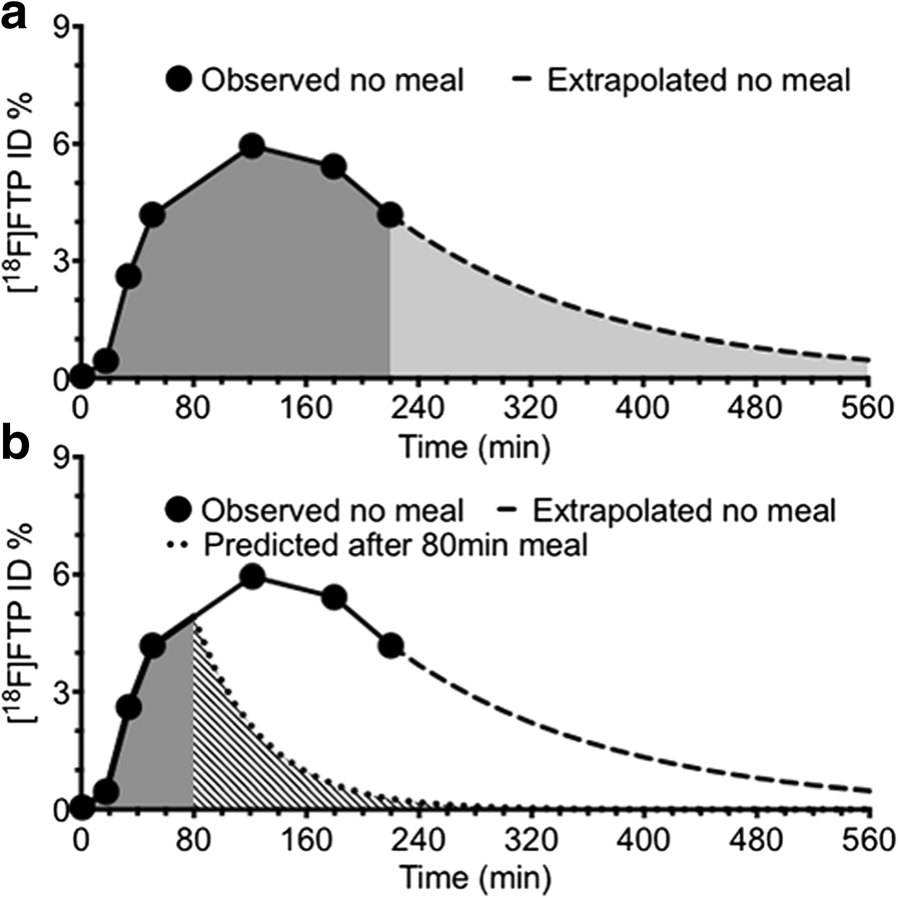
Subject 8’s gallbladder dose without a meal (**a**) and simulated dose with a fatty meal (**b**). Legend: Example of the gallbladder dose in an individual volunteer without a fatty meal (**a**) and simulated gallbladder dose for the same patient after consumption of a fatty meal at 80 min to match this subject’s intervention consumption time (**b**) where the extrapolated no meal curve is based upon an exponential decay fit to the last two experimental data points. The dark gray area under the curve represents experimentally collected data, whereas the light gray shaded area in **a** represents the predicted portion of the dose. The striped area under the curve in **b** illustrates the predicted dose using the fatty meal simulation. Difference in total shaded areas under the curve in **a** and **b** represent a change in dose received by the gallbladder with the addition of a fatty meal

**Fig. 4:**
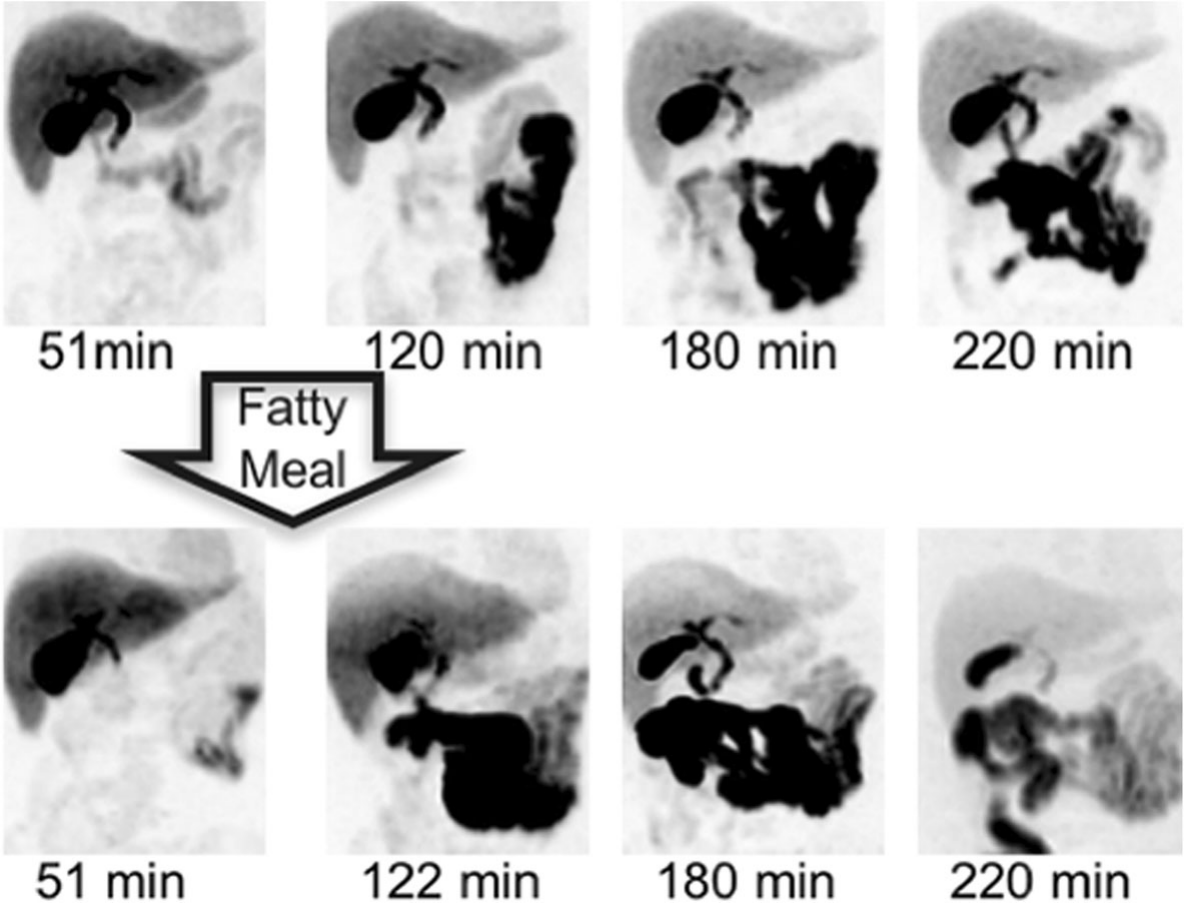
Maximum intensity pixel PET images for volunteer 8 in both control and intervention groups. Legend: The upper row of images shows the original scans, while the lower image set details the gallbladder contraction over time following a fatty meal during the volunteer’s repeat scan

The gallbladder clearance curve for a volunteer in the control group is shown in Fig. 3a, while the simulated clearance curve for the same volunteer in the simulation group is shown in Fig. 3b. To validate the simulations, the intervention group redid the study with consumption of a fatty meal during the 43 ± 11 min between the fourth and fifth serial PET scans at approximately 90 min after injection of radiotracer. This resulted in gallbladder contraction as illustrated in Fig. 4. Gallbladder contraction resulted in a new biodistribution of radioactive dose shown in Table 2.

**Table 2:** Mean FTP radiation dose estimates for the intervention group (mSv/MBq, *n* = 4)

Target organ	Mean	Standard deviation
Adrenals	1.34E−02	8.91E−04
Brain	4.37E−03	7.43E−04
Breast	6.11E−03	6.25E−04
Gallbladder wall	1.25E−01	8.41E−02
Lower large intestine wall	5.04E−02	1.12E−02
Small intestine	1.32E−01	3.19E−02
Stomach wall	1.40E−02	4.80E−04
Upper large intestine wall	1.51E−01	3.63E−02
Heart wall	1.93E−02	2.17E−03
Kidneys	2.57E−02	3.58E−03
Liver	5.76E−02	8.28E−03
Lungs	2.93E−02	1.22E−02
Muscle	8.84E−03	1.49E−04
Ovaries	2.70E−02	4.78E−03
Pancreas	1.54E−02	9.68E−04
Red marrow	1.04E−02	5.51E−04
Osteogenic cells	1.02E−02	3.39E−04
Skin	5.37E−03	1.96E−04
Spleen	3.09E−02	1.11E−02
Testes	5.84E−03	2.15E−04
Thymus	7.03E−03	7.49E−04
Thyroid	5.43E−03	4.62E−04
Urinary bladder wall	1.57E−02	4.16E−03
Uterus	2.14E−02	3.07E−03
Total body	1.22E−02	3.92E−04
Effective dose equivalent	4.40E−02	2.48E−03
Effective dose	2.67E−02	3.13E−03

Actual fatty meal consumption by the intervention group was 88 ± 9 min after injection of FTP and resulted in large changes in the biodistribution for specific regions as shown in Table 3.

**Table 3:** Comparing control, simulation, and intervention groups (mSv/MBq)

Target organ	Control	Simulation	Intervention	Percent change: control vs. intervention
Gallbladder	0.436	0.142	0.125	− 71.3
Upper large intestine	0.100	0.125	0.151	51.0
Lower large intestine	0.034	0.042	0.050	47.1
Small intestine	0.087	0.109	0.132	51.7
Effective dose	0.023	0.025	0.027	17.4

**Fig. 5:**
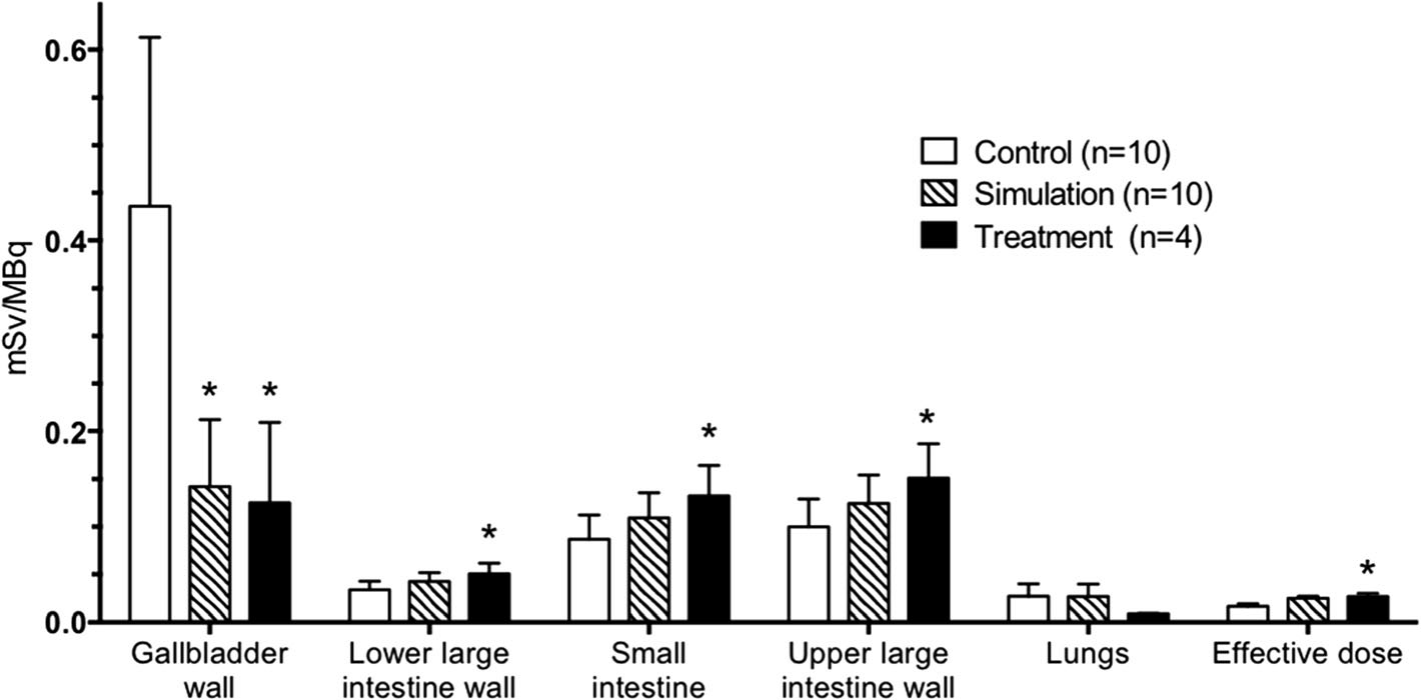
Comparisons of regional doses between control, simulation, and intervention groups. Legend: Asterisks indicate groups whose mean regional absorbed radiation doses are significantly different from the control group for that region using Tukey HSD analysis (*P* ≤ 0.027). Note that simulation and intervention groups were not significantly different for any of the regions shown (*P* ≥ 0.329)

ANOVA testing comparing the biodistribution of radiation dose of the control group (*n* = 10), the simulation group (*n* = 10), and the intervention group (*n* = 4) indicated significant differences between the groups for several regions including gallbladder, lower large intestine wall, small intestine, upper large intestine wall, and effective dose (*P* ≤ 0.028). Post hoc analysis was conducted using Tukey honest significant difference in order to determine the specific between-group differences for regions of interest. There were also statistically significant changes in dose between the control and intervention groups including the muscle, ovaries, pancreas, red marrow, osteogenic cells, testes, and uterus (*P* ≤ 0.046). The differences in doses were not significant (*P* ≥ 0.056) between the control and intervention groups for the other 13 regions (adrenals, brain, breasts, stomach wall, heart wall, kidneys, liver, lungs, skin, spleen, thymus, thyroid, and urinary bladder wall) in Tables 1 and 2. Figure 5 summarizes the key results of the between-group analyses.

## Discussion

Consumption of a fatty meal was predicted to redistribute radiotracer dose accumulating in the gallbladder by stimulating the gallbladder to contract and expel dose into the intestines as observed in Figs. 4 and 5. The simulation and intervention groups were not significantly different for the key regions reported in Fig. 5, indicating the model can accurately predict the change in biodistribution due to a fatty meal for the target organs of interest.

The high dose to the gallbladder may be due to in part to FTP’s relatively high lipophilicity (log *P* = 4.67 [15]). Lipophilicity, the tendency of a compound to diffuse into lipid-rich areas, has been associated high hepatic uptake and subsequent hepatobiliary excretion [24]. This is likely due to bile being composed of lipids as well as lipophilic bile acids [25]. As a result, FTP’s lipophilicity may play a role in its accumulation in the gallbladder. The gallbladder concentrates and stores bile during a fasting state [26], which is likely another factor causing the high gallbladder uptake due to participants being required to fast at least 4 h before imaging to minimize differences in dopamine levels due to consumption of food.

The significant difference in effective dose between the control and intervention groups in Fig. 5 was not expected because dose was only being redistributed between organs. This difference is due, in part, to the dose calculation method of OLINDA | EXM v1.1. For the gallbladder, the tissue weighting factor, a value proportional to the amount of effective dose each organ contributes to the total, is zero in this software version. This zero weight factor is based on the gallbladder not being included in the list of organs explicitly assigned by the International Commission on Radiological Protection (ICRP) to be included in effective dose calculations in the ICRP 60 Table S-2 [27]. The dose moving out of the gallbladder and into a weighted organ (i.e., intestines) increased the effective dose for both the simulation and intervention groups by now including radiation dose that was previously censored in effective dose calculations for the control group. The increase in effective dose following both simulation and intervention with fatty meal consumption appears to be a combination of an artifact of the calculation method’s zero gallbladder tissue weighting factor and higher radiosensitivity of the intestinal tissue. Future effective dose calculations should include a weighting factor for the gallbladder.

Theranostic PET radiotracers, which combine diagnostic and therapeutic capabilities into a single agent, may also benefit from application of the proposed gallbladder dose reduction model. Present theranostic agents, such as ^177^Lu-based compounds targeting somatostatin receptor and prostate-specific membranous antigen (PSMA) expressing metastatic disease, are mostly metabolized in the kidneys [28, 29]. However, development of theranostic PET agents that are hepatically eliminated are anticipated in the near future and may subject the gallbladder to significant, undesirable radiation burden. The validated simulation presented in this study provides the construct to overcome this obstacle through the informed ingestion of a fatty meal.

## Conclusions

[^18^F]Fluortriopride PET dosimetry simulations and experimentally validated results confirm a significant reduction in gallbladder radiation dose from PET radiotracers by employing intrinsic physiology to contract the gallbladder following an imaging session using an inexpensive fatty meal. Patients should consume a fatty meal (e.g., 8 oz can of Ensure Plus) following an FTP-PET scan to reduce dose to the gallbladder by up to 71% from 0.436 ± 0.177 mSv/MBq to 0.125 ± 0.0841 mSv/MBq. An injected dose of 240.5 MBq yielded an effective dose of 6.5 mSv for the intervention group and confirmed expectations that biodistribution and levels of absorbed doses were adequately low to continue researching FTP radiotracer uptake in humans. We recommend feeding all participants an inexpensive fatty meal following PET or SPECT imaging or theranostic sessions if the radiotracer or radioactive treatment has slow hepatobiliary clearance to reduce potentially high gallbladder radiation dose.

## Abbreviations

*A*: Estimated gallbladder activity
ANOVA: Analysis of variance
*A*_*o*_: Initial gallbladder activity at time of fatty meal consumption
CT: Computed tomography
*D*: Total estimated number of disintegrations following meal consumption
*D*_*o*_: Number of disintegrations occurring at the time of meal consumption
Fatty meal: 8 oz can of Ensure Plus from Abbott Laboratories
FTP: [^18^F]Fluortriopride
HSD: Tukey honest significant difference
ICRP: International Commission on Radiological Protection
*n*: Group sample size
oz: Ounce
PET: Positron emission tomography
*t*: Time in hours after consumption of fatty meal
VOI: Volume of interest
